# Benign paroxysmal positional vertigo, dizziness, and health-related quality of life among older adults in a population-based setting

**DOI:** 10.1007/s00405-020-06357-1

**Published:** 2020-09-19

**Authors:** Ellen Lindell, Lena Kollén, Mia Johansson, Therese Karlsson, Lina Rydén, Hanna Falk Erhag, Hanna Wetterberg, Anna Zettergren, Ingmar Skoog, Caterina Finizia

**Affiliations:** 1 Department of Otorhinolaryngology, Head and Neck Surgery, Institute of Clinical Sciences, Sahlgrenska Academy, Sahlgrenska University Hospital, Gothenburg University, 413 45 Göteborg, Sweden; 2 Region Västra Götaland, Department of Otorhinolaryngology, Sahlgrenska University Hospital, 413 45 Göteborg, Sweden; 3 Department of Occupational Therapy and PhysiotherapyInstitute of Clinical Sciences, Sahlgrenska Academy, Gothenburg University, 413 45 Göteborg, Sweden; 4 Department of Oncology, Institute of Clinical Sciences, Sahlgrenska Academy, Gothenburg University, 413 45 Göteborg, Sweden; 5 Neuropsychiatric Epidemiology Unit, Institute of Neuroscience and Physiology, Sahlgrenska Academy, Centre for Ageing and Health (AgeCap) at the University of Gothenburg, Göteborg, Sweden

**Keywords:** Dizziness, HRQL, Aged, BPPV, Quality of life, Walking speed

## Abstract

**Purpose:**

Dizziness may affect quality of life in a negative way and contribute to falls. The aim of this study was to investigate and compare 75 years old with dizziness caused by benign paroxysmal positional vertigo (BPPV) to those with general dizziness/impaired balance (non-BPPV related) and to those reporting no dizziness, regarding health-related quality of life (HRQL), falls, tiredness, and walking speed in a population-based setting.

**Method:**

A cross-sectional population-based sample, including 671 75 years old (398 women, 273 men), was investigated for BPPV, dizziness symptoms, falls, and walking speed. HRQL was assessed using the 36-item Short Form-36 Health Survey (SF-36).

**Result:**

A total of 67 persons (10%) had symptoms of BPPV with 11 (1.6%) having nystagmus when tested for BPPV. Having BPPV as well as general dizziness/impaired balance was associated with reduced HRQL, more tiredness, enhanced number of falls, and lower walking speed. Furthermore, the risk of having BPPV increased fourfold if symptoms of dizziness when turning in bed was reported.

**Conclusion:**

Having problems with dizziness is common among senior citizens where BPPV can be an unrecognized cause of dizziness that may impact HRQL and overall well-being. As BPPV is common among older adults, and is potentially curable through reposition maneuvers, it is important to liberally test for, and treat the condition. Information about dizziness when turning in bed can help to pinpoint persons with enhanced risk for BPPV also on a population-based level.

## Introduction

Dizziness is a common complaint among older adults, and is, therefore, often regarded as a normal age-related phenomenon or as part of aging itself. Problems with dizziness or unsteadiness are reported by approximately one-third of adults over 70 years, and becomes even more frequent with increasing age [1]. Dizziness may have multiple etiologies, but vestibular causes are especially common [2]. Dizziness and impaired balance can also be due to multiple morbidities and general weakness of all balance enhancing systems [3]. Benign paroxysmal positional vertigo (BPPV) is the single most common cause of dizziness from the vestibular system and is caused by otoconia from the utricle entering the semi-circular canal [4, 5]. The clinical characteristics of BPPV are dizziness and vertigo, together with nystagmus provoked by positional changes, like lying down or turning over in bed [6, 7]. BPPV among older adults, however, tends to cause unsteadiness rather than the spinning sensation often seen in younger adults [8, 9]. Because of milder symptoms, older adults seem to adapt or adjust to the condition and BPPV may be found unrecognized until tested for [10, 11]. BPPV may, however, still create a sensation of unsteadiness and of having impaired balance and can, in the majority of cases, be treated. Hence, treatment of the condition is highly recommended [5].

Patients with typical symptoms of BPPV, but without nystagmus when tested, are well described in the literature and also included in the consensus document of the Barany Society [4]. The magnitude of nystagmus during testing is supposed to be due to the size of the otoconial particles remaining in the semicircular canal [12]. The absence of positional nystagmus may, therefore, be due to the presence of only few particles; too little to produce nystagmus but enough to produce mild symptoms of dizziness, which is the most supported theory [12, 13]. Patients with BPPV without nystagmus also benefit from repositioning maneuvers [14–16]. The term subjective BPPV has been suggested for the condition with findings of typical dizziness during testing but where no objective nystagmus can be detected [4, 13, 15].

Health-related quality of life (HRQL) refers to the self-perceived health status or the functional abilities of the person [17]. It is the person’s subjective experience that relate both directly and indirectly to health, disease, and disability. Symptoms of dizziness and impaired balance have been negatively associated with HRQL [18–20] and patients with prolonged symptoms of dizziness tend to report higher morbidity and poorer HRQL. Treatment of BPPV effectively reduces symptoms of dizziness and also improves HRQL [21, 22].

Injuries caused by falling among older adults are common and as many as 32–42% of persons over 70 experience at least one fall annually [23]. The most frequently reported causes of falls are accidental or environmental causes, but dizziness, balance disorder, as well as difficulties in transferring are listed as second most common causes [24]. Vestibular dizziness, such as BPPV and general dizziness/impaired balance regardless of reason, may contribute to falls [24, 25].

Walking speed is a reliable and valuable test to measure general health [26]. A normal walking speed is an indicator for good health and level of fitness. Dizzy persons tend to limit their walking speed and walk more slowly than persons without dizziness [27, 28]. Walking speed > 1 m/s reflects good health, a good functional status, and overall well-being [29, 30].

Prior reports have mainly focused on BPPV patients in balance clinics. To our knowledge, fewer reports have been made regarding occurrence of BPPV, as well as useful questions to predict examination findings for unrecognized BPPV, on a population-based level and BPPV in relation to dizziness not synonymous with BPPV. The 75 years old in this study have previously been described and analyzed for BPPV [31]. However, BPPV has not been investigated in relation to HRQL, tiredness, and health, or in relation to general dizziness/impaired balance.

The aim of this study was to investigate and compare 75 years old with dizziness caused by.

BPPV to those with general dizziness/impaired balance (non-BPPV related) and to those reporting no dizziness, regarding HRQL, falls, tiredness, and walking speed in a population-based setting.

## Method

### Study population

The study is part of the population-based Gothenburg H70 birth cohort studies, Sweden. In this study, a cohort of 75 years old living in Gothenburg and born in 1930 was examined in 2005. The sample was selected through systematic sampling based on date of birth using the Swedish national population register [32], and included both persons living in ordinary and special housing. The present study has a cross-sectional design, and data were collected by medical doctors, trained research nurses, and physiotherapists at the neuropsychiatric outpatient clinic at the Sahlgrenska University hospital, or in the participants’ home. A total of 1295 persons (783 women, 512 men), all aged 75, were eligible for invitation to the multidisciplinary study and 841 participated (response rate 65%). A total of 671, 398 women and 273 men were included in this study, as they answered questions regarding dizziness. The design, procedures, and methods for overall data collection in the H70 studies have been described in detail elsewhere [33].

### Side-lying test for testing BPPV

The side-lying test was used for testing for BPPV. The participant was sitting on a bed and had their head turned, pointing 45° to the side. With the head in this position, the person was moved from the sitting position to be lying on the side that was to be tested. The person was informed to keep their eyes open, so possible nystagmus could be detected. The lying position was maintained for 2 min and the participant was then returned to sitting position and the same procedure was performed on the other side [34]. Rotational dizziness/vertigo was coded as yes or no. If dizziness occurred while testing with side-lying test; the occurrence of nystagmus was noted. A person was categorized as having BPPV if getting positional dizziness and/or having nystagmus in the side lying the test. Frenzel´s goggles were not used during the test. A total of 571 persons underwent the test.

### Dizziness, health, and tiredness

Study-specific measures contained questions about dizziness, general health, and diseases. The questions concerning dizziness were phrased: ‘‘Do you have any problems with dizziness or impaired balance?” (seldom/ sometimes/frequently), “Do you experience unsteadiness when walking?” (seldom/sometimes/frequently), “Do you have problems with vertigo/dizziness when turning in bed or bending backwards/forwards?” (seldom/sometimes/frequently). Concerning self-reported health and tiredness; the questions “Do you feel healthy?” (yes/no) and “Do you feel generally tired?” (yes/no) were asked.

### HRQL: Short Form Health Survey questionnaire (SF-36)

The 36-item Short Form-36 Health Survey (SF-36) is one of the most used tools for measuring HRQL and has proven useful in monitoring health, estimating burdens of diseases as well as evaluating effects of medical treatment [35], and was used to investigate HRQL. The test consists of 36 questions distributed over eight subscales: physical function (PF), role limitation due to physical problems (RP), bodily pain (BP), general health (GH), vitality (VT), social function (SF), role limitation due to emotional problems (RE), and mental health (MH) [35]. Every component is standardized and the scores range from 0 to 100 where low scores represent low HRQL. Validity and reliability have been evaluated and confirmed in prior studies, and the Swedish version of SF-36 has been validated in a representative sample of the population [36]. A total of 665 participants answered the SF-36 questionnaire.

### Walking speed and falls

Walking speed was tested over 20 m at self-selected and maximum speed, with no acceleration or deceleration phase. A total of 541 participants completed the walking tests. The study-specific measures included the following questions about falls:”Have you fallen during the last year? (yes/no)” and “How many times have you fallen?”.

### Groups

The participants were categorized into three groups: (1) participants with BPPV (dizziness and/or nystagmus in the side lying position during the test), (2) participants reporting having problems with dizziness/impaired balance sometimes or frequently but negative side lying test, and (3) participants without problems with dizziness.

### Statistical analyses

Test of differences between groups included Fisher´s exact test for dichotomous variables and Mantel–Haenszel Chi-square for ordered variables and *t* tests for continuous variables. To find questions indicative for having BPPV, logistic regression analyses were performed and relative risk calculations were performed. The result was presented as odds ratios with 95% confidential interval. Significance was always reported for two-tailed tests and the significance level used was 5%. The software used for the statistical analysis was SPSS, and a statistics program package developed at the Department of Geriatrics at Gothenburg University (GIDSS for Windows).

## Results

### Dizziness symptoms and BPPV

A total of 67 persons (10%) had BPPV with positional symptoms of rotational dizziness/vertigo and/or nystagmus in the side-lying test, also previously reported [31]. Of these 67 participants, 11 (16%) had nystagmus during the test and can be considered having definite BPPV according to the criteria from the Barany society [4]. A total of 201 reported general problems of dizziness or impaired balance (frequently or sometimes), but had no signs of positional symptoms of dizziness during testing with the side lying test. No symptoms of dizziness or impaired balance in combination with a negative side-lying test was reported by 403 individuals.

Problems with positional symptoms of dizziness when turning or bending over were common among those with BPPV with nystagmus (55%) as well as among those with BPPV-type dizziness (44%) but also among those with general dizziness/impaired balance (35%), compared to only 2% among those without complaints of dizziness (Table 1). Participants reported no difference in symptoms if having positional symptoms of dizziness during the test with or without detectable nystagmus present (Table 1). Subgroup analyses of participants with BPPV symptoms and nystagmus (*n* = 11) separately did not yield further significant results when compared to the group of participants without dizziness or when compared to the group with general dizziness. Therefore, these two BPPV groups were merged and labeled only BPPV for further analysis. The proportion of having BPPV if reporting dizziness when tilting the head or turning in bed (i.e., the positive predictive value) was 28% opposed to the proportions of 6% if not reporting dizziness when turning in bed. The sensitivity for the question of dizziness by turning in bed being was 45% (95% CI 33–58) and the specificity 84% (95% CI 81–88). The odds ratio of having BPPV compared to having either general dizziness/impaired balance or no dizziness if experiencing dizziness when tilting the head or turning over in bed was 5.6 (95% CI 3.2–9.6, *p* < 0.001) and the relative risk 4.3 (95% CI 2.8–6.7, *p* < 0.001).

**Table 1:** Symptoms of BPPV, dizziness, falls, health, tiredness, and walking speed of persons’ dizziness/impaired balance and no dizziness

	BPPV Nystagmus (*n* = 11)	BPPV No nystagmus (*n* = 56)	*p* value^a^	General dizziness/Impaired balance (*n* = 201)	No dizziness (*n* = 403)	*p* value^b^	*p* value^c^
Women *n* (%)	8 (67)	39 (70)		127 (63)	224 (56)		
Men *n* (%)	3 (33)	17 (30)		74 (37)	179 (44)		
Dizziness by turning in bed or leaning forward/backward [*n* (%)]							
Rarely	5 (45)	30 (56)	0.4	130 (65)	395 (98)	0.1	< 0.001
Sometimes/Frequently	6 (55)	24 (44)		70 (35)	8 (2)		
Unsteadiness when walking [*n* (%)]							
Rarely	4 (36)	32 (59)	0.1	76 (38)	367 (91)	< 0.05	< 0.001
Sometimes/Frequently	7 (64)	22 (41)		123 (62)	35 (9)		
Previously falling last 12 months [*n* (%)]	2 (18)	19 (36)	0.3	69 (38)	86 (23)	0.6	ns
Number of falls							
0	9 (82)	33 (63)	0.4	112 (62)	287 (77)	0.2	0.013
1	1 (9)	10 (19)		35 (19)	68 (18)		
2–3	1 (9)	7 (13)		29 (16)	15 (4)		
> 3	0 (0)	2 (4)		5 (3)	3 (1)		
Feeling generally tired [*n* (%)]	3 (27)	18 (35)	0.7	53 (29)	37 (10)	0.5	< 0.001
Not feeling well [*n* (%)]	1 (9)	10 (19)	0.7	37 (20)	29 (8)	0.2	0.031
Walking speed				*n* = 149	*n* = 335		
Walking 20 m, maximal speed, mean m/s (SD)	1.54 (0.29)	1.51 (0.32)	0.8	1.57 (0.34)	1.71 (0.18)	0.5	< 0.001
Walking 20 m, normal speed, mean m/s (SD)	1.09 (0.19)	1.11 (0.19)	0.7	1.13 (0.21)	1.21 (0.18)	0.2	< 0.001

*p* values based on Fisher´s exact test (unsteadiness when walking, dizziness when turning in bed, previously falling, feeling tired, not feeling well), Mantel–Haenzel Chi-square test (number of falls) and t test (walking speed)

^a^Difference between BPPV with and without nystagmus

^b^Difference between BPPV (with and without nystagmus analyzed together as one group, *n* = 67) and general dizziness/impaired balance

^c^Difference between BPPV (with and without nystagmus analyzed together as one group, *n* = 67) and no dizziness

When comparing only with those reporting no dizziness, the odds ratio for having BPPV if experiencing dizziness when tilting the head or turning over in bed was 41 (95% CI 17–96, *p* < 0.0001). Unsteadiness when walking was most prominent among those with general dizziness/impaired balance, with 62% reporting symptoms of unsteadiness, compared to 44% among those with BPPV and 9% of those without dizziness (Table 1). The odds ratio of feeling unsteadiness when walking was 8 (95% CI 4–14, *p* < 0.0001) if having BPPV and 17 (95% CI 10–26, *p* < 0.0001) if having general dizziness/impaired balance compared to not having dizziness. Participants with BPPV and general dizziness/impaired balance were more tired and felt less well than non-dizzy participants (Table 1). The odds ratio of feeling tired was 3.9 (95% CI 2.5–6.1, *p* < 0.0001) if having BPPV compared to not having dizziness. The corresponding figure of not feeling well was 2.8 (95% CI 1.8–4.7, *p* < 0.001).

### HRQL and tiredness

Having BPPV or general dizziness/impaired balance was negatively associated with HRQL. There was no difference in HRQL between participants with BPPV compared to those with general dizziness/impaired balance except for the domain role physical, where the BPPV group had better values and similar scores to participants without dizziness (Table 2). There were no significant differences in HRQL between participants with BPPV with or without nystagmus why these two groups were analyzed together.

**Table 2:** Health-related quality of life of persons with BPPV, general dizziness/impaired balance, and no dizziness

	BPPV (*n* = 67)	General dizziness/impaired balance (*n* = 196)	No dizziness (*n* = 401)	*p* value^a^	*p* value^b^
SF-36	Mean (SD)	Mean (SD)	Mean (SD)		
Physical functioning	69.3 (20.8)	63.7 (22.5)	77.8 (20.8)	ns	0.004
Role physical	72.2 (37.5)	56.1 (41.1)	75.5 (36.8)	0.004	ns
Bodily pain	64.0 (25.1)	59.3 (23.9)	74.7 (24.6)	ns	0.003
General health	62.0 (19.2)	59.1 (18.9)	71.6 (17.5)	ns	< 0.001
Vitality	60.1 (20.9)	59.2 (20.3)	72.6 (20.1)	ns	< 0.001
Social functioning	82.9 (22.2)	82.2 (21.3)	91.1 (17.2)	ns	0.002
Role emotional	71.6 (37.7)	73.5 (37.8)	83.4 (32.1)	ns	0.013
Mental health	71.4 (20.6)	75.3 (19.3)	83.7 (19.7)	ns	< 0.001

The maximum score for SF-36 is 100 for every domain with higher scores which indicate better HRQL

*p* values are based on *t* test

^a^Difference between BPPV and general dizziness/impaired balance

^b^Difference between BPPV and no dizziness

### Falls

Persons with BPPV did not fall more than others. However, when number of falls during the previous year was categorized into four groups: 0, 1, 2–3, and > 3 falls, participants with BPPV as well as those with general dizziness/impaired balance had a higher frequency of falls (Table 2). Having problems with general dizziness/impaired balance was also associated with a higher risk of falls and odds ratio 2.1 (95% CI 1.4–3.0, *p* < 0.001) compared to not having dizziness.

### Walking speed

The participants without dizziness walked faster than those having BPPV or those having dizziness/impaired balance at both maximum and normal speed (Table 1). The difference was more prominent at maximum speed. No difference in walking speed between people with BPPV compared to those with general dizziness/impaired balance was observed.

## Discussion

We found a high frequency of dizziness and a 10% occurrence of BPPV-type dizziness in this population-based sample of 75 years old. Identifying reasons for dizziness in older people can be difficult, yet important as some disorders, like BPPV can be managed to lessen the symptoms. We found that the proportion having BPPV if reporting dizziness when turning in bed was 28% compared to 6% if not reporting dizziness when turning in bed. The risk of having BPPV increased fourfold if experiencing dizziness when tilting the head or turning over in bed, compared to those reporting no dizziness. Dizziness (both BPPV and general dizziness) was associated with lower quality of life, higher reports of tiredness and not feeling well, higher number off falls, and lower walking speed in this population-based sample of 75 years old. Worth to notice is that only 11 (16%) of those having positive side-lying test had nystagmus during the test. However, in participants experiencing dizziness during positional testing, there was no difference regarding reported dizziness when turning in bed, unsteadiness when walking, falls, reported tiredness and health, and walking speed between participants having nystagmus or no identifiable nystagmus. However, it is possible that a larger study population would have yielded more statistically significant results.

Dizziness when tilting the head or turning in bed are common complaints among patients with BPPV seeking medical care for their dizziness [6]. Here, we present a cohort of 75 year old from the general population, not patients, and still, reporting dizziness by positional changes might help to identify persons with higher risk for BPPV, emphasizing that the phenomenon of dizziness when turning in bed can be a useful tool when identifying persons with BPPV, also among adults in the community not seeking care for their symptoms.

BPPV can most often easily be treated with canalith reposition maneuvers (CRM), such as Epley´s or Semont´s [37]. Also, patients with symptoms of BPPV but without eye motor findings may benefit from treatment with CRM [13, 16]. Huebner et al. showed significant improvement of symptoms measured with the Dizziness handicap inventory scale after treatment with CRM advocating for providing treatment even for persons without objective findings of BPPV [16]. Lopez-Escamez et al. showed that treatment of BPPV with particle reposition maneuvers improved HRQL, especially in the physical domains [38]. It is, therefore, of great importance for every clinician meeting older people with balance problems and dizziness to have knowledge of the BPPV testing and treatment options. Older adults may also adapt to the condition and avoid quick head movements, why testing for and treating BPPV should be performed liberally even if no typical history exist.

Having BPPV as well as other types of dizziness were associated with a number of negative outcomes, such as lower HRQL, worse self-reported health, more tiredness, lower speed of gait and higher number of falls. The questionnaire SF-36 is a tool evaluating HRQL in both physical and psychological aspects and much focus is put on function. It is therefore interesting to ponder to what extent *function* in this age group equals HRQL? Having dizziness was associated with a reduction in HRQL and therefore all potential treatment options should be considered to reduce symptoms of dizziness and thereby possibly enhance overall well-being.

Having BPPV was not associated with falling at least one time during the last year. However, participants with BPPV, as well as those with general dizziness/impaired balance, had a greater risk of having experienced several falls and thereby having an enhanced risk of suffering fall-related injuries as falls among older adults may have devastating consequences. Persons with dizziness also had a lower walking speed than those without dizziness. Experiencing fear of falling itself may be a reason for being careful and not moving fast to avoid falls [39]. Tiredness was also more common among persons with dizziness than in those without; one reason for this could be that a substantial amount of energy is invested in maintaining balance and avoid falling.

Training programs, like weight training and balance and functional exercises, may be effective to reduce the rate of falls and potentially also to enhance balance [40]. However, the evidence for general exercise to improve balance among older adults in general is weak [41]. Hopefully, vestibular training can be a way to improve balance and lessen the dizziness symptoms [42]

In our study, the prevalence of BPPV diagnosed through the side-lying maneuver was high, but the frequency of nystagmus during the test was substantially lower. One probable reason for this is that we did not use Frenzel´s glasses or goggles with infrared video camera, making nystagmus more difficult to detect. According to the criteria of the Barany society, the definite diagnosis of BPPV requires symptoms of dizziness and canal-specific nystagmus when tested with positional maneuvers. However, one should be aware that positional nystagmus detectable with videonystagmography, but no dizziness, is common among healthy adults [43]. Information regarding typical symptoms of dizziness during positional testing is, therefore, of great importance, especially in a population-based setting, since over-diagnosing BPPV might otherwise be a risk.

### Strengths and limitations

Strengths of our study include the representative population-based sample and that the examinations were performed by health professionals. The study is one of the few studies investigating BPPV on a large population-based level, even if not all completed the side lying test. However, there are also some limitations to consider. Due to the cross-sectional design, the direction of causality is not possible to establish. Frenzel’s glasses or videonystagmography was not used in the testing for BPPV, making nystagmus more difficult to detect. The result for the side-lying test would have been more precise if goggles or infrared cameras had been used. Lack of visible nystagmus during positional changes may also be due to small quantity of otoconia, enough to trigger dizziness but not to get the response of nystagmus. The Dix–Hallpike’s test, often considered to be the gold standard for a diagnosis of pBPPV, was not used due to the high age of the participants, and in addition, the side-lying test was considered easier to perform and, therefore, used.

## Conclusion

Having problems with dizziness is common among senior citizens, where BPPV can be an unrecognized cause of dizziness which may impact HRQL and overall well-being. As BPPV is common among older adults and is potentially curable through reposition maneuvers, it is important to liberally test for and treat the condition. Information about dizziness when turning in bed can help to identify persons with enhanced risk for BPPV also on a population-based level.
